# Livers with Constitutive mTORC1 Activity Resist Steatosis Independent of Feedback Suppression of Akt

**DOI:** 10.1371/journal.pone.0117000

**Published:** 2015-02-03

**Authors:** Heidi L. Kenerson, Savitha Subramanian, Rebecca McIntyre, Machiko Kazami, Raymond S. Yeung

**Affiliations:** 1 Northwest Liver Research Program, Department of Surgery, University of Washington, Seattle, Washington, United States of America; 2 Division of Metabolism, Endocrinology and Nutrition, Department of Medicine, University of Washington, Seattle, Washington, United States of America; University College London, UNITED KINGDOM

## Abstract

Insulin resistance is an important contributing factor in non-alcoholic fatty liver disease. AKT and mTORC1 are key components of the insulin pathway, and play a role in promoting *de novo* lipogenesis. However, mTORC1 hyperactivity *per se* does not induce steatosis in mouse livers, but instead, protects against high-fat diet induced steatosis. Here, we investigate the *in vivo* mechanism of steatosis-resistance secondary to mTORC1 activation, with emphasis on the role of S6K1-mediated feedback inhibition of AKT. Mice with single or double deletion of *Tsc1* and/or *S6k1* in a liver-specific or whole-body manner were generated to study glucose and hepatic lipid metabolism between the ages of 6–14 weeks. Following 8 weeks of high-fat diet, the *Tsc1-/-;S6k1-/-* mice had lower body weights but higher liver TG levels compared to that of the *Tsc1-/-* mice. However, the loss of *S6k1* did not relieve feedback inhibition of Akt activity in the *Tsc1-/-* livers. To overcome Akt suppression, *Pten* was deleted in *Tsc1-/-* livers, and the resultant mice showed improved glucose tolerance compared with the *Tsc1-/-* mice. However, liver TG levels were significantly reduced in the *Tsc1-/-;Pten-/-* mice compared to the *Pten-/-* mice, which was restored with rapamycin. We found no correlation between liver TG and serum NEFA levels. Expression of lipogenic genes (*Srebp1c*, *Fasn*) were elevated in the *Tsc1-/-;Pten-/-* livers, but this was counter-balanced by an up-regulation of *Cpt1a* involved in fatty acid oxidation and the anti-oxidant protein, Nrf2. In summary, our *in vivo* models showed that mTORC1-induced resistance to steatosis was dependent on S6K1 activity, but not secondary to AKT suppression. These findings confirm that AKT and mTORC1 have opposing effects on hepatic lipid metabolism *in vivo*.

## Introduction

The growing epidemic of NAFLD is becoming a leading cause of chronic liver disease in the United States [1]. NAFLD refers to a spectrum of hepatic disorders secondary to an increase in hepatic triglyceride (TG) content in the setting of the metabolic syndrome that is associated with obesity, hypertension, hyperlipidemia and type II diabetes [2]. Steatosis is a necessary step in the pathogenesis of NAFLD, but inflammation is required to bring about steatohepatitis, which leads to chronic damage to hepatocytes and progressive fibrosis. Dietary control and exercise are the mainstay for prevention and treatment of NAFLD, while long-term pharmacologic interventions have not been adopted.

Insulin promotes lipid synthesis and storage while repressing glucose synthesis in the liver. In patients with metabolic syndrome, the liver becomes selectively resistant to insulin with respect to gluconeogenesis but remains responsive to lipogenesis [3,4]. Recent studies have identified key components of the insulin pathway, namely Akt, mTORC1 and S6K1, to play positive roles in promoting *de novo* lipogenesis [5]. Each of these 3 kinases converges on SREBP1c to enhance its function as a master transcription factor in coordinating the expression of enzymes involved in lipid synthesis.

Experiments in genetically modified livers highlight the central role of Akt in promoting lipogenesis. The loss of hepatic *Pten* leads to profound steatosis that is dependent on Akt2 activity [6,7] [8]. Acting downstream of Akt, mTORC1 phosphorylates lipin-1 to promote SREBP1c nuclear localization and activity such that in the absence of raptor, a defining component of mTORC1, diet-induced *de novo* lipogenesis is suppressed [9]. In addition, S6K1 plays a role in lipogenesis by promoting SREBP1c processing [10] [11]. Interestingly, others and we have found that mTORC1 hyperactivity per se was insufficient to induce steatosis in mouse livers. Instead, mice with hepatocyte-specific deletion of *Tsc1* were resistant to high-fat diet induced steatosis [12,13]. In the absence of *Tsc1*, Akt activity is suppressed through multiple mechanisms [14], which may explain the findings in the *Tsc1*-/- livers. Specifically, mTORC1 activation negatively feeds back to PI3K by S6K1 phosphorylation of IRS1 and by regulating Grb10 stability via direct phosphorylation [15–18]. In addition, the loss of TSC1-TSC2 complex impedes mTORC2 phosphorylation of Akt(Ser473) [19]. We have shown previously that transient over-expression of myristylated Akt in the *Tsc1*-/- livers led to accumulation of TG [12]. Yecies et al. suggested that a target of Akt, Insig2, may be responsible for reduced SREBP1c processing in the *Tsc1*-/- livers [13]. However, the *in vivo* effects of mTORC1-induced negative feedback on hepatic lipid metabolism remain unclear.

In this study, we used genetic models to examine the role of S6K1 and Akt in protecting *Tsc1*-/- livers against steatosis. We found that S6K1 activity mediates mTORC1-induced suppression of steatosis independent of Akt feedback inhibition. Further, co-activation of Akt and mTORC1 in livers deleted of both *Pten* and *Tsc1* failed to accumulate TG despite an up-regulation of lipogenic gene expression. These findings suggest that the key kinases involved in insulin signaling do not function cooperatively in hepatic lipid metabolism. Instead, Akt and mTORC1 exert opposing effects to maintain hepatic lipid homeostasis.

## Materials and Methods

### Mice

All experiments were performed in accordance with the Institutional Animal Care and Use Committee (#3051) at the University of Washington, Seattle. *Tsc1^fl/fl^;Alb^cre^* and *Pten^fl/fl^;Alb^cre^* mice, as described [12] [20], were crossed to generate *Tsc1^fl/+^;Pten^fl/+^;Alb^cre^* mice, and, in turn, intercrossed to derive experimental mice with desired genotypes (*Tsc1^fl/fl^;Pten^+/+^;Alb^cre^, Tsc1^+/+^;Pten^fl/fl^;Alb^cre^, Tsc1^fl/fl^;Pten^fl/fl^;Alb^cre^*) within the same genetic background. Littermates not carrying the Cre alleles were used as controls. Similarly, *Tsc1^fl/fl^;Alb^cre^* were crossed with *S6k1-/-* (full body knockout, gift of W. Laidges, UW) mice to generate *Tsc1^fl/+^; Alb^cre^ S6k1+/-* mice, and, in turn, intercrossed to derive experimental mice with desired genotypes (*Tsc1^fl/fl^; Alb^cre^;S6k1+/+, Tsc1^+/+^;Alb^cre^;S6k1-/-*, and *Tsc1^fl/fl^;Alb^cre^;S6k1-/-*) within the same genetic background. Littermates not carrying the *Cre* alleles that were *S6k1*+/+ were used as controls. Mice were fasted overnight before death by CO_2_ inhalation. For rapamycin treatment, mice were treated with 2mg/kg of rapamycin (Calbiochem) for 2 weeks.

### Histology

Slides were deparaffinized, rehydrated, and washed before staining with hematoxylin QS and eosin (Vector Laboratories, Burlingame, CA) and mounting with Permount (Fischer Scientific, Santa Clara, CA). For Oil Red O staining, 5 micron thick frozen sections of livers were cut and stained with Oil Red O diluted in propylene glycol.

### Metabolic studies

At six weeks of age cohorts of control, *Tsc1*-/-, *S6k1*-/- and *Tsc1*-/-;*S6k1*-/- mice were placed on either normal chow diet (NCD) (PicoLab Rodent Diet 20, 5053, LabDiet) composed of 25% protein, 13% fat, and 62% carbohydrate, or a high fat diet (HFD), (AIN-76A, 58R2, TestDiet) composed of 15% protein, 25.7% carbohydrate, 59.3% fat for eight weeks. Mice were weighed weekly.

Glucose Tolerance Test (GTT) was performed in mice fasted for sixteen hours after an intraperitoneal (IP) injection of 10% glucose (1 mg/g body weight). Blood glucose was measured at 0, 15, 30, 60, and 120 minutes in blood obtained via tail nicking using the OneTouch glucometer (LifeScan, Inc., Milpitas, CA). Similarly, insulin sensitivity testing was performed after a 4-hour fast and 0.5 mU/g IP injection of regular insulin (Humulin R, Eli Lilly) followed by glucose measurements at 0, 30, 60, 90, and 120 minutes.

### Western blotting

Mouse livers were homogenized in ice-cold radioimmunoprecipitation (RIPA) buffer (1% Nonidet P-40, 1% sodium deoxycholate, 0.1% SDS, 0.15 M NaCl, 10 mM Tris (pH 7.2), 0.025 M β-glycophosphate (pH 7.2), 2 mM EDTA, and 50 mM sodium fluoride) with protease and kinase inhibitors (0.05 mM AEBSF, 10 μg/ml aprotinin, 10 μg/ml pepstatin, 1 mM orthovanadate, 10 μg/ml leupeptin, 1 mM microcystin LR). The protein concentration was measured using the BCA Protein Assay (Pierce, Rockford, IL). Equal amounts of protein were separated by SDS-PAGE, transferred to Immobilin-P membranes (Millipore, Bedford, MA) and blotted with antibodies according to manufacturer recommendations. Nrf and ATF4 antibodies were purchased from Abcam (Cambridge, MA) and Santa Cruz Biotechnology, Inc. (Dallas, TX) respectively. The FGF21, and p62 antibodies were purchased from Sigma (St. Louis, MO). All other antibodies were purchased from Cell Signaling (Danvers, MA).

### qRT-PCR

Total RNA was extracted from ~100 mg liver tissue using a commercially available RNA extraction kit according to the manufacturer’s protocol (Agilent Technologies, Santa Clara, CA). After spectroscopic quantification, 2 μg of RNA was reverse-transcribed, and cDNA was analyzed by real-time quantitative PCR. Primers specific for individual genes were purchased from Invitrogen or IDT. All data were normalized to housekeeping gene GAPDH. Relative amounts of the target gene were calculated using the ΔΔCt formula.

### Metabolic Parameters

Blood was extracted via cardiac puncture immediately after sacrifice. Blood was spun for 15 minutes at 3000 rpm at 4°C. Plasma triglycerides (TG) were measured using a colorimetric assay kit and plasma insulin was measured using a commercially available ELISA kit (Millipore, Billerica, MA). Plasma non-esterified fatty acid (NEFA) levels were analyzed from fresh plasma using a kit from Zen-Bio (Research Triangle Park, NC). Hepatic triglyceride content was determined following lipid extraction using the Folch method [21].

### Statistical Analyses

Results were illustrated by box-and-whisker plot of the median, where the box extends to the 25th and 75th percentiles and the whiskers were placed at the 5th and 95th percentiles (Graphpad Prism). Comparisons of quantitative data between two groups were analyzed using the Student t-test. For multi-group comparisons, analysis of variance (ANOVA) was used. Differences were considered significant at p-value of less than 0.05. Sample size ranged between 3 to 7 per group.

## Results

### Effects of high-fat diet in *Tsc1-/-;S6k1-/-* mice

We have previously shown that the steatosis-resistant phenotype in the *Tsc1*-/- liver was completely reversed by rapamycin treatment suggesting that mTORC1 activity prevents diet-induced steatosis [12]. Further, Akt activity was suppressed in the *Tsc1*-null livers, and acute Akt activation (via Myr-Akt expressing adenovirus) induced steatosis in the *Tsc1^fl/fl^;Alb^Cre^* mice [12]. The relationship between mTORC1 and Akt activities is regulated by mTORC1-dependent and-independent mechanisms [14]. Here, we focused on mTORC1/S6K1 feedback inhibition of Akt as a mechanism for the suppression of steatosis. We generated various mutant mice consisting of liver-specific ablation of *Tsc1* and/or whole-body deletion of *S6k1*. The *S6k1*-/- mice were previously shown to be resistant to diet-induced obesity [22], but their hepatic lipid phenotype has not been characterized.

**Figure 1:**
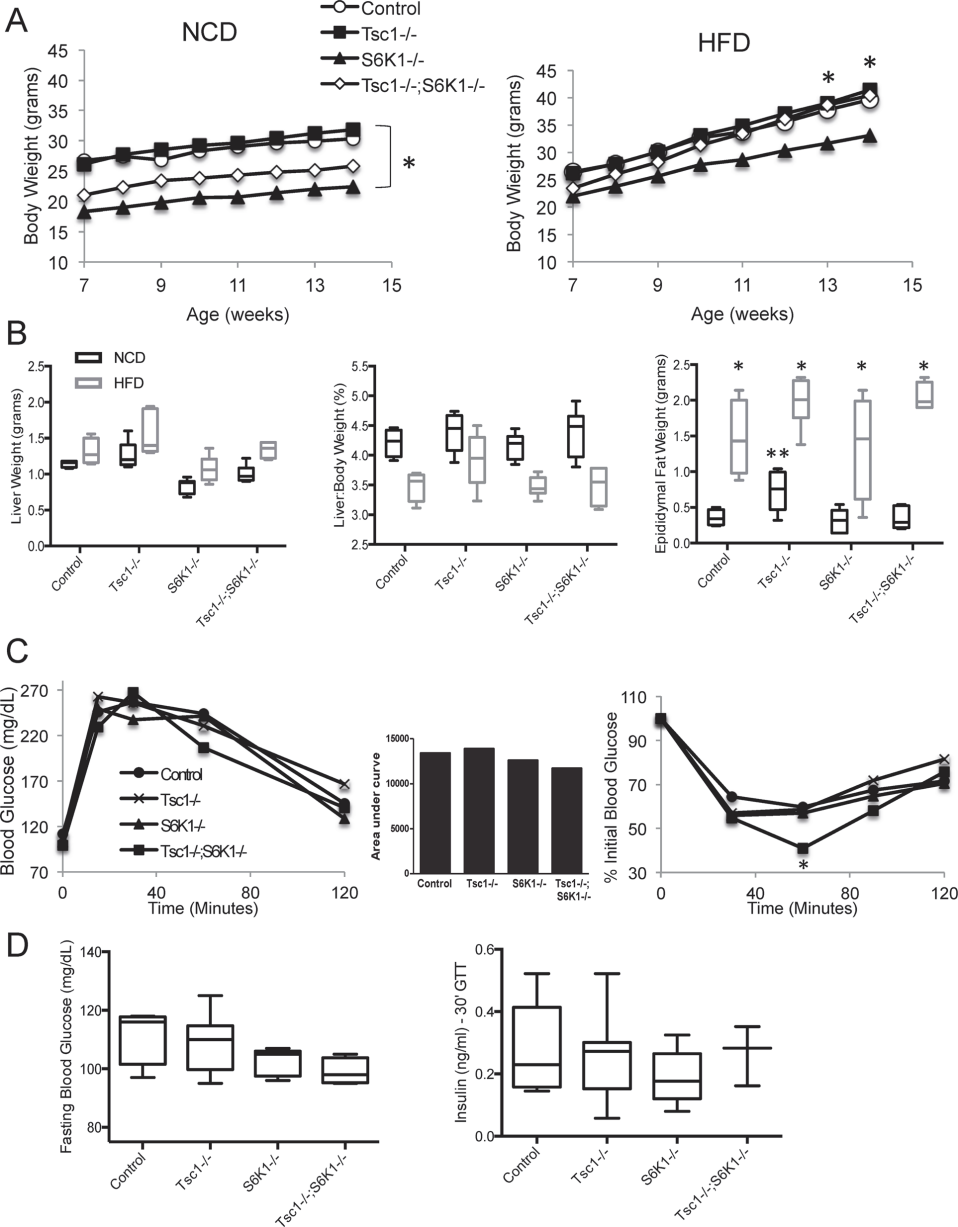
The *Tsc1^fl/fl^;Alb^Cre^;S6k1*-/- mice develop normally and have increased insulin sensitivity. A) Total body weights of male littermates fed either NCD or HFD over the study period. *, p<0.05 comparing *S6k1*-/- with *Tsc1*-/-. B) Box-and-whisker plots of liver weights (absolute and relative) and epididymal white adipose tissue weights following 8 weeks of NCD or HFD. *, p<0.05 compared to NCD within genotype, **, p<0.05 compared to control within diet. C) Glucose tolerance and insulin sensitivity tests. *, p<0.05 comparing *Tsc1*-/-;*S6k1*-/- to control. D) Fasting blood glucose and plasma insulin levels 30 minutes following glucose administration. N = 4–7 mice per group.

The *Tsc1^fl/fl^;Alb^Cre^;S6k1*-/- mice were viable and developed normally. PCR and Western blot analyses confirmed liver-specific deletion of *Tsc1* and whole-body deletion of *S6k1* (S1 Fig. and data not shown). To determine their susceptibility to weight gain, 6 wk-old male littermates were subjected to high-fat diet (HFD) for 8 weeks ad-libitum. Fig. 1A compares total body weights in mice fed normal chow diet (NCD) vs. HFD. Consistent with a previous study[22], the NCD-fed *S6k1*-null mice were smaller. However, relative weight gains over the 8-wk period were identical among all 4 groups while on NCD, and marginally reduced in the HFD-fed *S6k1*-null cohort, again in keeping with published report[22]. The corresponding liver weights in the NCD-fed *S6k1*-/- mice were also lower compared to controls, but increased significantly following HFD as did the *Tsc1^fl/fl^;Alb^Cre^;S6k1*-/- livers (Fig. 1B). HFD also led to a decrease in liver-to-body weight ratios and an increase in epididymal fat without significant differences between mice of various genotypes.

Glucose metabolism was evaluated by insulin sensitivity test and glucose tolerance test between 8 to 9 weeks of age. The results shown in Fig. 1C did not demonstrate significant differences between the four groups with the exception of significantly lower plasma glucose levels in the *Tsc1^fl/fl^;Alb^Cre^;S6k1*-/- mice 60 minutes following insulin injection. Corresponding insulin levels at 30 minutes following glucose injection and fasting blood glucose were not significantly different (Fig. 1D).

### Lipid accumulation in the *Tsc1-/-;S6k1-/-* liver occurs independent of Akt

**Figure 2:**
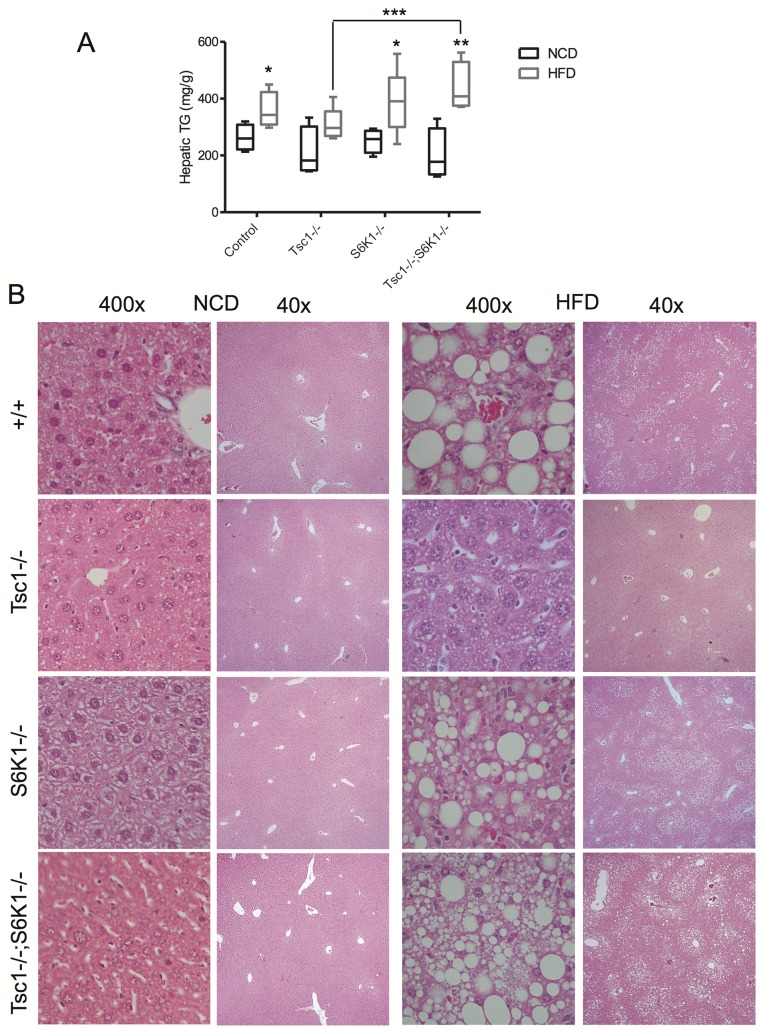
*S6k1* deletion promotes steatosis in *Tsc1*-null hepatocytes. A) Liver triglyceride (TG) levels in male mice fed NCD (black) and HFD (gray). *, p<0.05 compared to respective NCD; **, p<0.01 compared to respective NCD; ***, p = 0.03 comparing *Tsc1*-/- vs. *Tsc1*-/-;*S6k1*-/- on HFD. N = 4–7 mice per group. B) Examples of H&E histology of livers showing varying degrees of macrovesicular steatosis under NCD and HFD conditions. Original magnifications: 40x and 400x. Note the lack of large lipid droplets in HFD-fed *Tsc1*-/- livers.

Next, we assessed the extent of steatosis by measuring liver TG content and examining liver histology with H&E staining. When fed NCD, there were no significant differences in hepatic TG levels among the four genotypes (Fig. 2A), and the corresponding histology showed no evidence of macrovesicular steatosis (Fig. 2B, NCD). Following HFD, hepatic TG levels rose significantly in every group except in the *Tsc1*-/- mice (Fig. 2A). Importantly, we found significantly higher TG levels in the *Tsc1^fl/fl^;Alb^Cre^;S6k1*-/- livers compared to the *Tsc1*-/- livers under HFD condition. These biochemical results are confirmed by histologic analyses showing large lipid droplets in livers of all genotypes except for the *Tsc1*-/- livers under HFD condition (Fig. 2B, HFD)., These findings indicate that S6k1 suppresses steatosis in the setting of constitutive mTORC1 activation.

**Figure 3:**
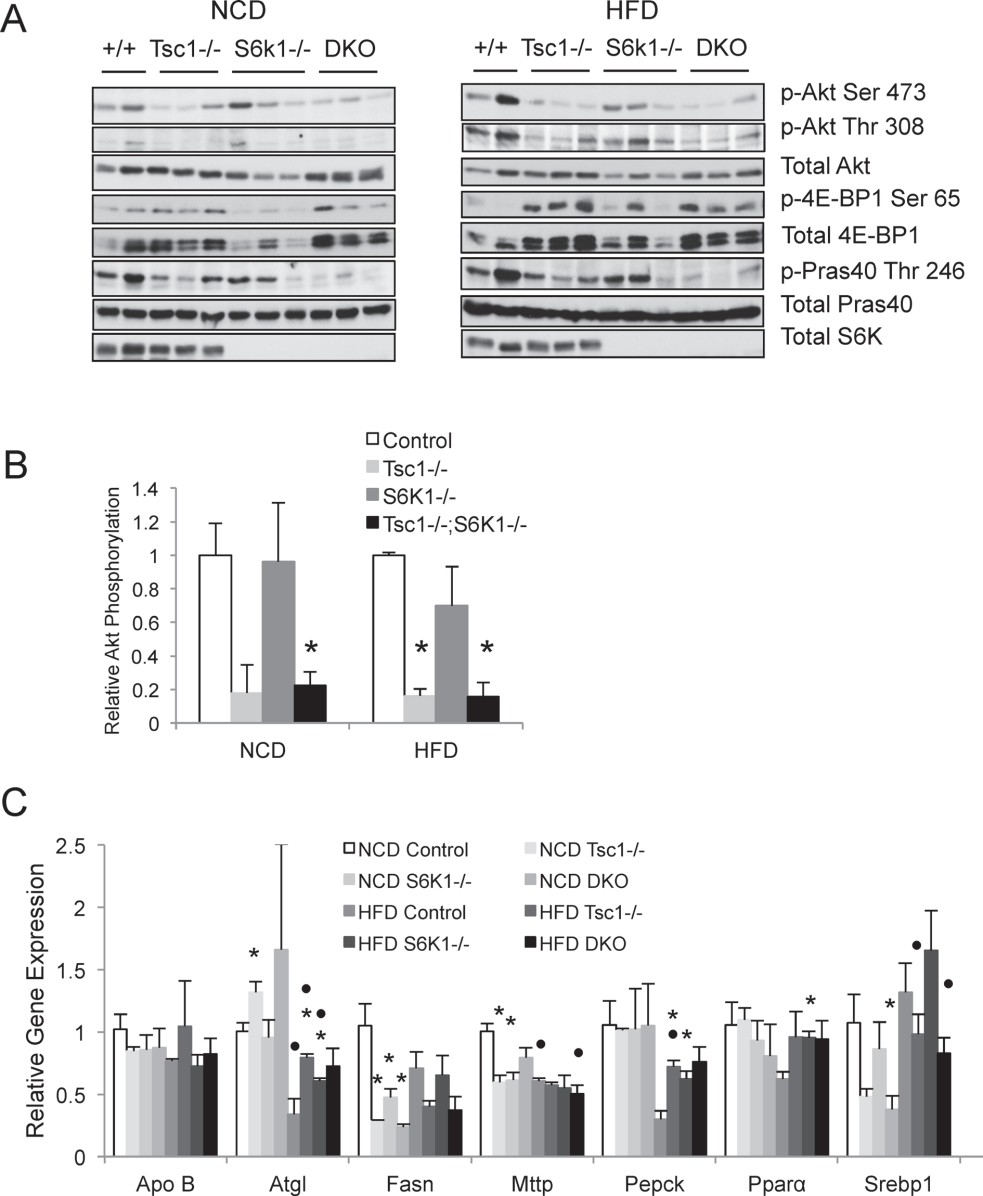
Deletion of *S6k1* in *Tsc1*-/- livers does not relieve Akt suppression. A) Akt and mTORC1 signaling in livers under NCD and HFD conditions. Akt activity is indicated by the phosphorylation of Ser473 and Thr308 and that of its substrate, Pras40(Thr246), whereas mTORC1 activity is reflected in 4E-BP1(Ser65) phosphorylation. Note that the *S6k1*-/- mice do not express S6k as expected. B) Graph shows levels of Akt phosphorylation relative to total Akt expression based on densitometry (Image J) of the blots shown in A. C) Expression of genes relevant to lipid metabolism based on qRT-PCR of indicated liver samples. *, p<0.05 compared to respective controls within diet (NCD or HFD); •, p<0.05 NCD of the same genotype. N = 4–7 mice per group.

To determine whether the effects of S6k1 loss on TG accumulation were secondary to feedback activation of Akt activity, we examined Akt-mTORC1 signaling in NCD- and HFD-fed livers using Western blot analyses. Fig. 3A shows that the *Tsc1*-/- livers under both dietary conditions had lower Akt activities (based on Akt and Pras40 phosphorylation) compared to control livers while phosphorylation of 4E-BP1(Ser65) was higher; these findings are consistent with mTORC1-mediated feedback suppression of Akt in the liver. In contrast, the *S6k1*-/- and *Tsc1^fl/fl^;Alb^Cre^;S6k1*-/- livers lost expression of S6k1 as a result of its genetic deletion, but phospho-4E-BP1(Ser65) expression was elevated in the ‘double mutant’ mice compared with *S6k1*-/- livers indicating that mTORC1 remained active in the *Tsc1^fl/fl^;Alb^Cre^;S6k1*-/- livers. We quantified relative hepatic Akt activities in these fasted livers based on the ratio of phospho-Akt(Ser473) to total Akt expression for each genotype using densitometry measurements and further normalized to the respective wild-type controls. Fig. 3B shows the pattern of relative Akt phosphorylation in the livers to be similar after 8 weeks of NCD and HFD. The expression of phospho-Pras40(Thr246) followed the same trend (data not shown). These findings indicate that the suppressed Akt activity observed in the *Tsc1*-/- livers did not significantly increase following deletion of *S6k1*, even when fed a HFD. Mice with *S6k1* deletion alone had similar levels of Akt phosphorylation compared to wild-type controls. Thus, the loss of *S6k1* was not sufficient to relieve the feedback inhibition of Akt caused by mTORC1 activation *in vivo*; this implies that other factor(s) is/are involved in mTORC1-mediated inhibition of Akt as previously discussed [14]. Our findings further indicate that S6k1 limits steatosis in the *Tsc1*-null livers through an Akt-independent mechanism. Consistent with this, the expression of lipogenic and other metabolic genes that typically accompany Akt-induced lipogenesis were not significantly different between the *Tsc1*-/- and *Tsc1^fl/fl^;Alb^Cre^;S6k1*-/- livers under HFD condition (Fig. 3C). Of note, the lipolytic enzyme, *Atgl*, was over-expressed in the *Tsc1*-/- livers compared to controls in both NCD and HFD groups, consistent with previous report [12], but the differences in the double mutant livers did not reached statistical significance.

### Combined loss of *Pten* and *Tsc1* in the liver does not result in steatosis

**Figure 4:**
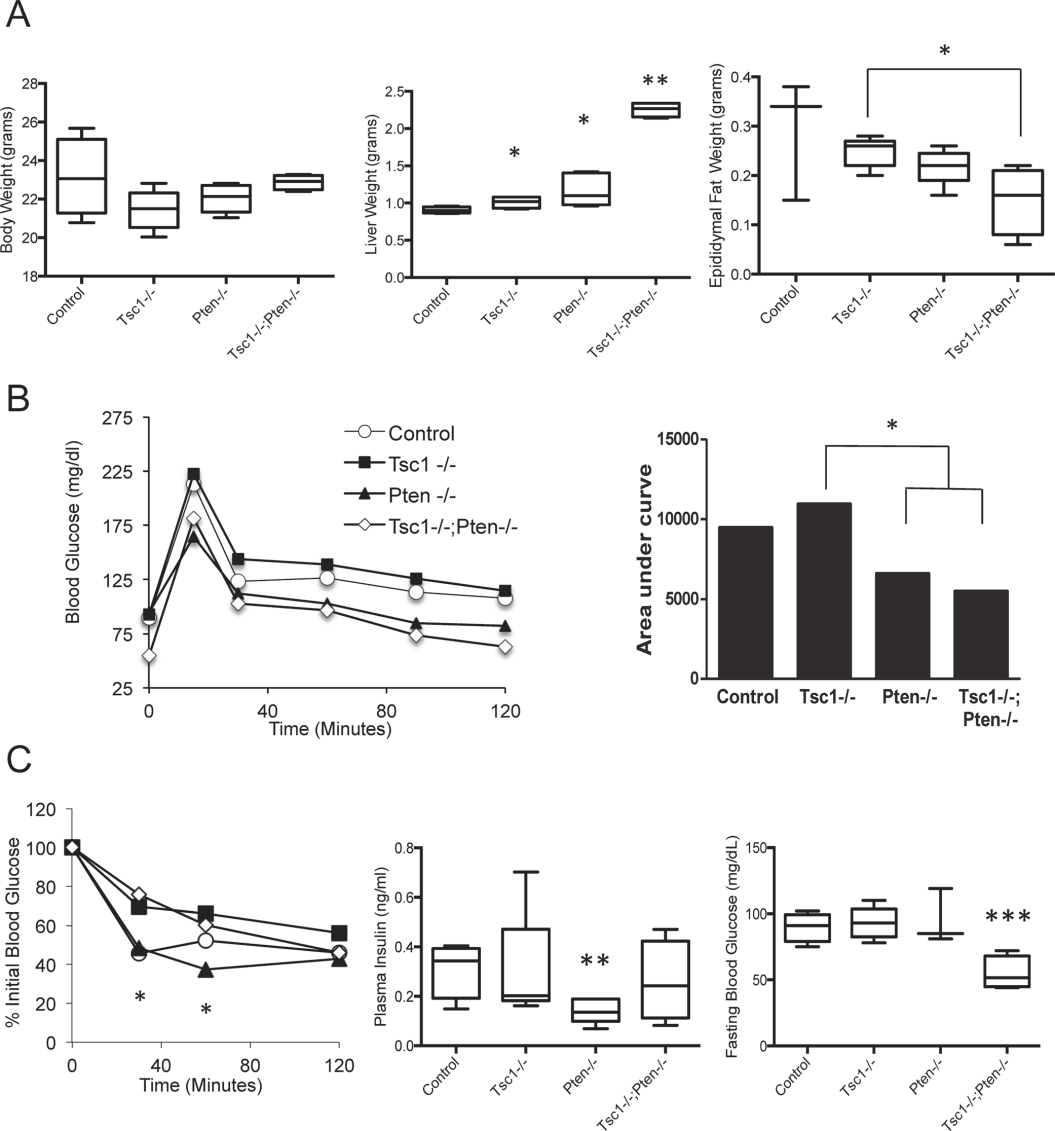
Co-deletion of *Pten* and *Tsc1* in hepatocytes leads to hepatomegaly and hypoglycemia. A) Comparisons of total body, liver and epididymal WAT weights. *, p<0.05 compared to control or where indicated by lines. **, p<0.0001 compared to all other groups. B) Glucose tolerance test with ‘area under the curve’ graph. *, p<0.05 comparing *Tsc1*-/- with *Pten*-/- and *Tsc1*-/-;*Pten*-/-. C) Insulin sensitivity test, fasting plasma insulin and fasting glucose levels. *, p<0.01 between *Pten*-/- and *Tsc1*-/-. **, p<0.01 compared to control. ***, p<0.01 compared to all groups. N = 3–6 mice per group.

To directly address the hypothesis that the lack of steatosis in the *Tsc1*-/- liver is a result of Akt suppression, we deleted the dual-specific phosphatase, *Pten*, in hepatocytes of the *Tsc1^fl/fl^;Alb^Cre^* mice to force Akt activation and overcome mTORC1-induced inhibition of PI3K/Akt. Genetic breeding of the *Pten^fl/fl^;Alb^Cre^* mice with the *Tsc1^fl/fl^;Alb^Cre^* mice produced viable *Tsc1^fl/fl^;Pten^fl/fl^;Alb^Cre^* (a.k.a. *Tsc1*-/-;*Pten*-/-) mice as previously described (see S1 Fig.) [20]. The *Tsc1*-/-;*Pten*-/- livers enlarged rapidly in the first few months and developed tumors by week 14. Thus, to minimize the influence of multi-focal tumors on liver function and metabolism, mice of all genotypes were studied at 10 weeks of age and were fed NCD. Even at this age, livers of the *Tsc1*-/-;*Pten*-/- mice were significantly larger than other genotypes despite similar body weights (Fig. 4A). The ‘double mutant’ mice also accumulated significantly less epididymal fat compared to the *Tsc1*-/- mice. Glucose tolerance test indicates improved glucose clearance among the *Pten*-/- and *Tsc1*-/-;*Pten*-/- animals compared to the *Tsc1*-/- mice consistent with previous observations (Fig. 4B) [12]. Similarly, the *Pten*-/- mice showed greater sensitivity to insulin compared to the *Tsc1*-/- animals (Fig. 4C). Correspondingly, fasting plasma insulin was reduced in the *Pten*-/- mice but unchanged in the *Tsc1*-/-;*Pten*-/- mice compared to control. Additionally, fasting glucose levels were significantly lower in the *Tsc1;Pten*-double mutants compared to other groups (Fig. 4C).

**Figure 5:**
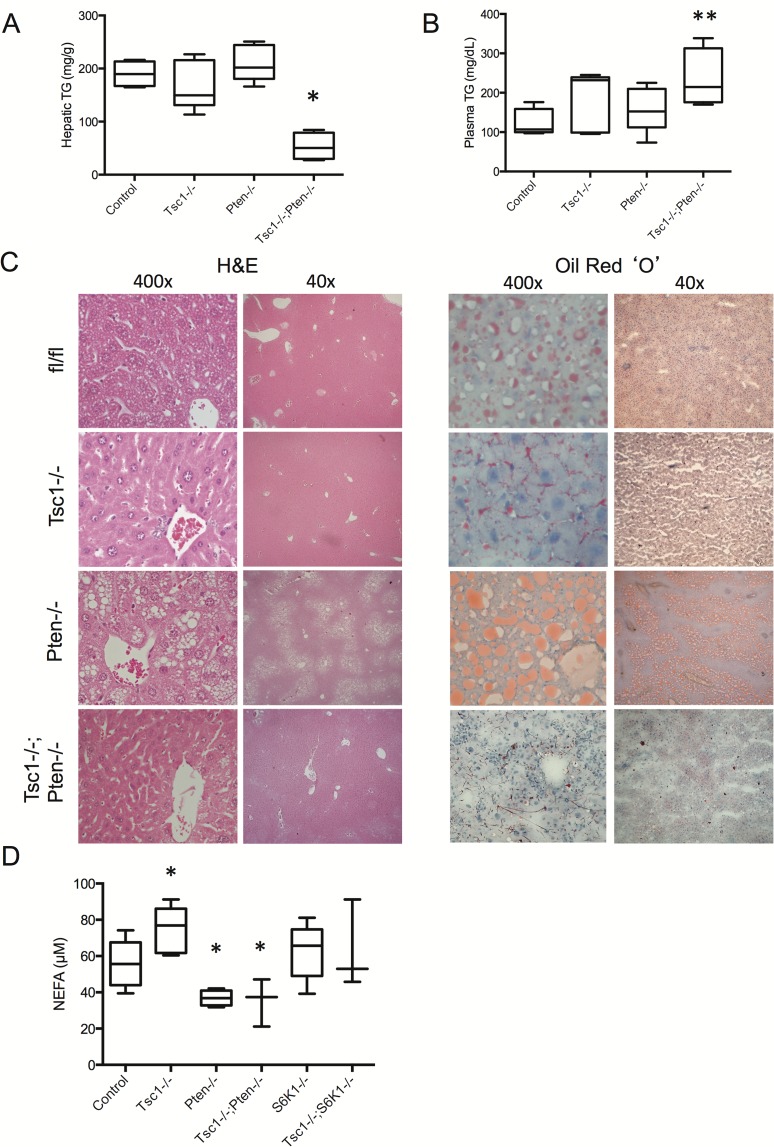
*Pten* deletion does not promote steatosis in the *Tsc1*-/- livers. A) Liver TG content and B) Plasma TG. *, p<0.01 compared to all other groups. **, p<0.05 compared to control. N = 4–6 mice per group. C) Liver histology by H&E and Oil red ‘O’ staining. Mice were fed NCD and fasted overnight before sacrifice. Original magnifications: 400x and 40x. fl/fl, floxed control. D) Non-esterified fatty acids levels in freshly drawn blood samples. *, p<0.05 compared with control group. N = 3–7 per group.

**Figure 6:**
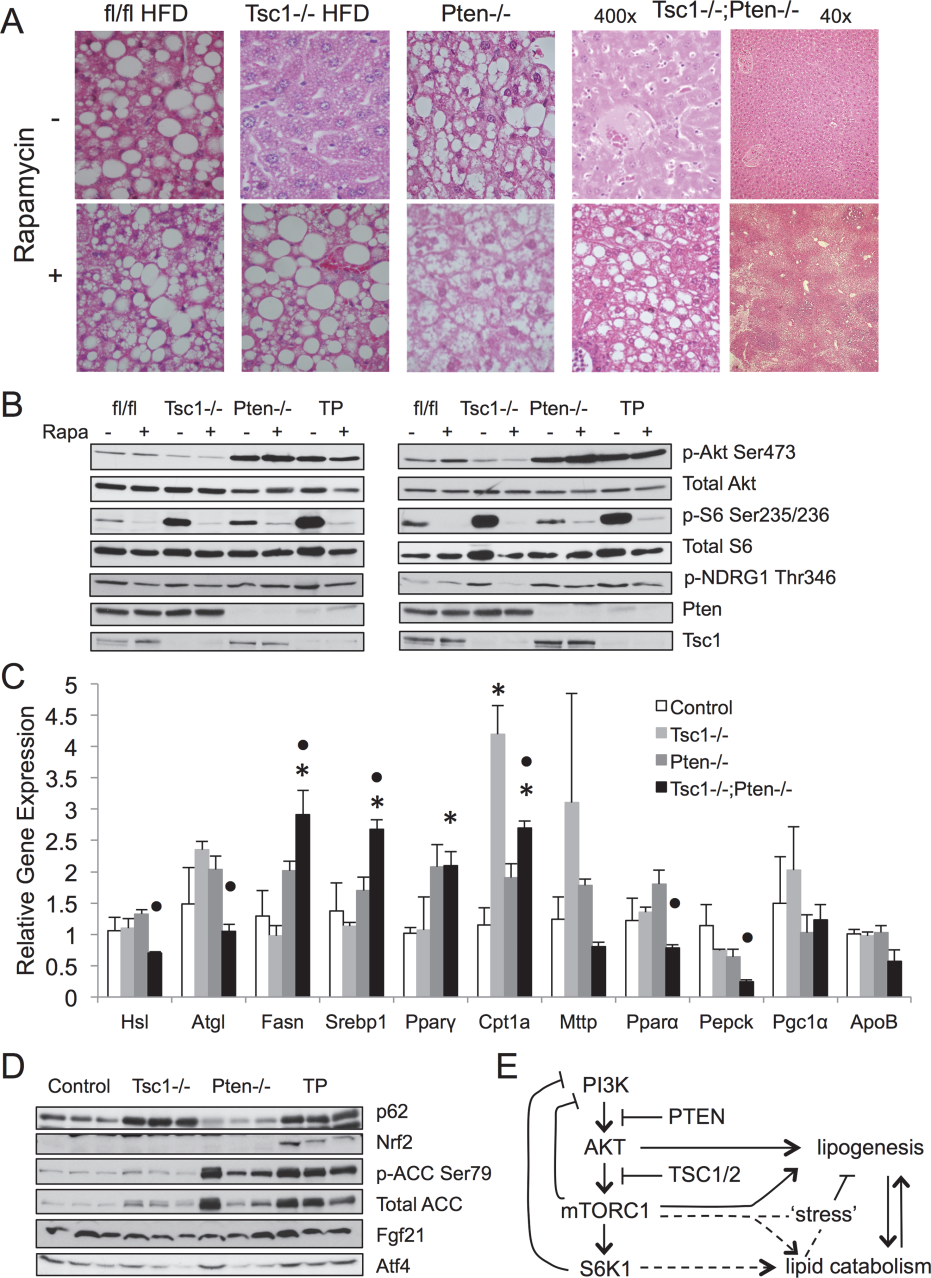
Rapamycin increases steatosis in *Tsc1*-/- and *Tsc1*-/-;*Pten*-/- livers. A) H&E photomicrographs of representative livers treated with vehicle (DMSO) or rapamycin. Magnification: 400x except for *Tsc1*-/-;*Pten*-/- samples showing both 40x and 400x. B) Western blot analyses of liver lysates from two sets of treated mice. Note lack of change in Akt phosphorylation following two weeks of rapamycin. mTORC2 activity is indicated by p-Akt(Ser473) and p-NDRG1(Thr346) expression. fl/fl, floxed controls. TP, *Tsc1*-/-;*Pten*-/-. C) Expression of genes relevant to lipid metabolism based on qRT-PCR of indicated liver samples. *, p<0.05 compared to control. •, p<0.05 compared to *Tsc1*-/-. N = 3–6 mice per group. D) Western blot analyses of markers of metabolic, energy and autophagic stress. E) Model highlighting the *in vivo* effects of Akt-mTORC1-S6K1 in lipid metabolism. See text for details.

At 10 weeks of age, hepatic TG content in the *Tsc1*-/-;*Pten*-/- livers was surprisingly lower than control livers while plasma TG levels were significantly elevated (Fig. 5A, B). This was confirmed by H&E histology and Oil Red ‘O’ staining highlighting the lack of macrovesicular steatosis in the *Tsc1*-/-;*Pten*-/- livers (Fig. 5C). At this young age (e.g., 10 wks), TG levels in the *Pten*-/- livers have not risen significantly over that of the control and *Tsc1*-/- livers. However, histologic evidence of steatosis can be found in the 10-week old *Pten*-/- livers, which was restricted to the peri-venule zone; this was not observed in mice of other genotypes (Fig. 5C). By 13–14 wks of age, we observed marked increase in liver weight and accumulation of TG in the *Pten*-/- livers (S2 Fig.) [12]. On the other hand, non-tumor regions of the *Tsc1*-/-;*Pten*-/- livers at 14 wks failed to show evidence of steatosis (upper panel, Fig. 6A). Western blot analyses confirmed the expected changes in Akt and mTORC1 activities in the respective groups (Fig. 6B). Specifically, the *Tsc1*-/-;*Pten*-/- livers exhibited co-activation of Akt and mTORC1. Together, these findings indicate that persistent Akt activation in the *Tsc1*-/- livers did not result in steatosis, and conversely, up-regulation of mTORC1 in the *Pten*-/- livers protected against Akt-induced steatosis.

Circulating non-esterified fatty acids (NEFA) is an important substrate for *de novo* lipogenesis in the liver. One possible explanation for the observed hepatic TG findings could be due to differing NEFA levels in mice of various genotypes. Indeed, plasma NEFA concentration measured from freshly drawn blood samples was significantly lower in the *Tsc1*-/-;*Pten*-/- mice compared to controls (Fig. 5D). However, NEFA levels were also reduced in the *Pten*-/- mice, but significantly increased in the *Tsc1*-/- mice, and unchanged in the *S6k1*-/- and *Tsc1*-/-;*S6k1*-/- mice compared to control. Overall, we did not find a correlation between circulating NEFA levels and hepatic TG content. Therefore, the effect of mTORC1-induced protection against steatosis is independent of the influence of circulating non-esterified fatty acids.

To further address whether the absence of steatosis in the *Tsc1*-/-;*Pten*-/- livers was due to mTORC1 activity, mice were treated with rapamycin (2mg/kg, ip, daily, 2 wk) and examined for evidence of steatosis. Fig. 6A shows that mTORC1 inhibition was sufficient to induce lipid accumulation in the 14-week-old *Tsc1*-/-;*Pten*-/- hepatocytes similar to its effects observed in the *Tsc1*-/- livers. This implies that the *Tsc1*-/-;*Pten*-/- liver is capable of developing macrovesicular steatosis, and it does so in a rapamycin-sensitive manner. In addition to mTORC1 inhibition, rapamycin can affect Akt activity in a dose-, time- and cell type-dependent fashion, in part through its influence on mTORC2. Here, we monitored Akt(Ser473) and NDRG1(Thr346) phosphorylation by immunoblot analyses as indicators of mTORC2 activity[23] [24]. Fig. 6B shows that rapamycin consistently inhibited mTORC1 activity (i.e., rpS6 phosphorylation) but without uniform changes in phospho-Akt(Ser473) or phospho-NDRG1(Thr346) expression. This is in keeping with reports of persistent Akt(Ser473) phosphorylation in cells following prolonged rapamycin treatment despite disassembly of mTORC2 [25]. Similarly, Huang et al. found that rapamycin treatment only partially restored insulin-induced Akt phosphorylation in the *Tsc2*-/- MEFs [19]; this has been attributed to the effects of autophosphorylation or other kinases. In our study, relatively low-dose rapamycin did not significantly change the levels of Akt(Ser473) phosphorylation, and therefore, the observed steatotic phenotype following rapamycin treatment cannot be attributed to the effects of Akt. Together, these findings support the conclusion that mTORC1 activation confers steatosis resistance independent of Akt activity.

Analysis of the genes involved in lipid metabolism by qRT-PCR revealed that the *Tsc1*-/-; *Pten*-/- livers expressed increased levels of pro-steatotic genes including *Srebp1c*, *Fasn* and *Pparγ* (Fig. 6C), but the absence of steatosis indicates the existence of counter-balancing factors. We previously reported elevated expression of the lipolytic genes, *Atgl*, in the *Tsc1*-/- livers [12], but this was not observed in the *Tsc1*-/-;*Pten*-/- livers. Further, genes involved in VLDL assembly and secretion (ApoB, Mttp) were not significantly different, but instead, *Cpt1a* expression was significantly increased in both the *Tsc1*-/- and *Tsc1*-/-;*Pten*-/- livers suggesting a potential role of increased fatty acid oxidation in limiting hepatic TG accumulation. In addition, autophagy was found to be inhibited in the *Tsc1*-/- and *Tsc1*-/-;*Pten*-/- livers resulting in the accumulation of p62. These metabolic ‘stresses’ were reflected in the up-regulation of the anti-oxidant protein, Nrf2, which has been associated with the suppression of lipogenesis (Fig. 6D). Concurrently, phospho-ACC(Ser79) expression indicative of AMPK activity was also elevated in the *Tsc1*-/-;*Pten*-/- livers along with ATF4 as a result of ER stress. A recent study suggested a role of the stress-related FGF21 in limiting steatosis in the *Tsc1*-/- hepatocytes, but we found no significant difference in FGF21 expression in fasted livers of various genotypes (Fig. 6D). Our observations are consistent with ‘fat’ suppressing factors, perhaps secondary to metabolic stresses, which protect the *Tsc1*-/-;*Pten*-/- livers from lipid accumulation.

## Discussion

AKT, mTORC1 and S6K1 are key components of the insulin signaling pathway, which becomes dysregulated in patients with metabolic syndrome. Non-alcoholic fatty liver disease is a manifestation of systemic insulin resistance, resulting in a spectrum of disorders ranging from simple steatosis to severe steatohepatitis and cirrhosis. Steatosis represents a requisite but not sufficient step for NAFLD. Understanding the molecular pathogenesis of steatosis will provide a scientific rationale for its prevention. Experimental evidence demonstrates direct links between AKT, mTORC1 and lipogenesis such that stimulation of both kinases has been shown to promote *de novo* lipid synthesis *in vitro*. Further, Akt activation results in steatohepatitis in murine livers, thus making the *Pten^fl/fl^;Alb^Cre^* mice a useful model of NASH [7] [6] [8]. However, constitutive activation of mTORC1 following *Tsc1* deletion surprisingly protects against diet-induced steatosis [12] [13]. We previously showed that the ‘fat-resistant’ phenotype in the *Tsc1*-/- model was completely reversed with rapamycin treatment implicating a specific role of mTORC1 [12]. Yet, mice with liver-specific deletion of raptor, an essential component of mTORC1, have variable response to high fat diet [9]. A study by Peterson et al. showed the *raptor*-deficient livers to be resistant to diet-induced steatosis [9], while Umemura et al. reported a normal steatotic response to HFD in *raptor*-/- livers [26]. We previously found that *Pten*-null livers remained steatotic following rapamycin treatment. Thus, mTORC1 appears to be neither necessary nor sufficient for steatosis *in vivo*, and uncontrolled mTORC1 activity initiates compensatory mechanisms that protect the liver from excessive TG accumulation.

The most pervasive explanation for the ‘steatosis-protective’ phenotype in the *Tsc1*-null livers is the mTORC1 feedback inhibition of Akt. Previously, we showed that acute over-expression of Myr-Akt induced steatosis in the *Tsc1*-/- hepatocytes [12]. In this study, we used a genetic approach to investigate the role of S6K1 as a mediator of the negative feedback. We found that the deletion of *S6k1* in the *Tsc1*-null livers restored steatosis under HFD conditions. However, hepatic Akt activity in the *Tsc1/S6k1* ‘double’ mutants fed either NCD or HFD diet remained suppressed compared to wild-type controls. These findings indicate that S6K1 limits TG accumulation in the *Tsc1*-/- hepatocytes through an Akt-independent pathway (Fig. 6E). We have reported that genes involved in lipid catabolism are up-regulated in the *Tsc1*-/- livers [12], but the relationship between S6K1 and lipid utilization or other mechanisms of lipid suppression has not be elucidated.

The lack of Akt ‘re-activation’ upon *S6k1* deletion in the *Tsc1*-/- livers further implies other mechanisms of Akt suppression. The cross-talk between Akt and mTORC1 involves multiple feedback loops [14]. Besides S6K1/IRS1-mediated inhibition of PI3K signaling, phosphorylation of Grb10 by mTORC1 also blunts insulin signaling [17,18]. In addition, the absence of the TSC1-TSC2 complex has been shown to dampen Akt(Ser473) phosphorylation by mTORC2 [19]. It is unclear which of these mechanisms may be regulating Akt activity in the *Tsc1*-/-;*S6k1*-/- livers. Nonetheless, the lack of Akt activation in the steatotic livers of these mice led us to reconsider the role of Akt and mTORC1 in hepatic lipid metabolism. We had anticipated that the combined activation of Akt and mTORC1 in hepatocytes would cause an exaggerated accumulation of TG given the known effects of these kinases on lipogenesis [9]. To our surprise, co-deletion of *Tsc1* and *Pten* in livers resulted in the least amount of TG compared to control and single mutants. Since *Pten* deletion in the *Tsc1*-/- hepatocytes overcame Akt suppression, we conclude that Akt activity per se is insufficient to promote TG accumulation in hepatocytes with mTORC1 activation. We further showed that the lack of steatosis was due to mTORC1 since rapamycin treatment of the *Tsc1^fl/fl^;Pten^fl/fl^;Alb^Cre^* livers led to macrovesicular steatosis. These findings support the notion that mTORC1 protects against Akt-mediated steatosis, and that hepatic Akt activates lipogenesis independent of mTORC1. The latter is consistent with the observations made in a liver-specific rictor deletion model that harbors defective mTORC2 but intact mTORC1 signaling [27]. Collectively, our *in vivo* models indicate that Akt and mTORC1 have opposing effects on hepatic lipid accumulation, and that mTORC1 restricts steatosis through S6K1, independent of Akt suppression.

Both Akt and mTORC1 are known for their effects on cellular metabolism and are capable of inducing metabolic stress when constitutively activated resulting in ER stress, autophagy inhibition and AMPK activation. A recent study showed that the *Tsc1*-/- livers are protected from steatosis through ‘stress’-induced responses, including hepatic synthesis of FGF21 [28], but we were unable to find significant differences in FGF21 expression in livers of various genotypes. Instead, we noted an increase in Nrf2, a key transcription factor involved in antioxidant response, in the *Tsc1*-/-;*Pten*-/- livers. Nrf2 is known to limit lipogenesis and could play a role in these livers to minimize fat accumulation [29,30] [31] [32,33]. We further found elevated levels of *Cpt1a* transcripts in the *Tsc1*-/- and *Tsc1*-/-;*Pten*-/- livers, which can promote fatty acid oxidation. Together, it appears that a number of ‘compensatory’ mechanisms may provide protection against steatosis when Akt and mTORC1 are co-activated. Future studies will address these and other anti-steatotic mechanisms, which may have therapeutic implications.

In summary, our *in vivo* experiments highlight a protective role of mTORC1 in lipid homeostasis. While mTORC1 promotes SREBP1c activity in cells, persistent mTORC1 activity in hepatocytes protects against HFD-induced steatosis in a S6K1-dependent, Akt-independent manner. It further protects against AKT-induced steatosis. This *in vivo* effect of mTORC1 likely represents a compensatory mechanism that occurs when hepatocytes are placed under metabolic stress (Fig. 6E). The opposing roles of Akt and mTORC1 in hepatic lipid accumulation suggest that the degree of steatosis depends on the balance between Akt and mTORC1 activities. These findings predict that the manipulation of Akt and mTORC1 can be exploited to develop novel strategies in the treatment of fatty liver disease. Clinical evidence in support of this concept comes from reports of treatment-related steatosis in patients exposed to rapamycin analogs prescribed for cancer therapy [34]. In contrast, leucine and other branched-chain amino acids are potent activators of mTORC1 [35], and dietary supplements with these amino acids have been reported to alleviate NAFLD and its associated metabolic syndrome [36]. Direct evaluation of clinical samples from human steatotic and normal livers will provide further insights into the clinical significance of our findings.

## Supporting Information

S1 Fig Immunoblot analyses of Tsc1, Pten and S6k1 proteins in liver and adipose tissues from mice of various genetic crosses: C, fl/fl control; T, *Tsc1^fl/fl^ Alb^cre^;* P, *Pten^fl/fl^;Alb^cre^*; S, *S6k*1-/-; TP, *Tsc1^fl/fl^;Pten^fl/fl^;Alb^cre^*; TS, *Tsc1^fl/fl^; Alb^Cre^;S6k1*-/-. (TIFF)

S2 Fig Comparison of 10- and 13-week old *Pten^fl/fl^; Alb^cre^* livers showing significant increase in liver mass and triglyceride (TG) content over a 3 week period. At 10 weeks of age, TG contents were similar between the *Pten*-/- and control livers, but the difference became significant by 13 weeks of age. *, p<0.05; **, p<0.01.
(TIFF)
